# Genome Sequence of Canine Polyomavirus in Respiratory Secretions of Dogs with Pneumonia of Unknown Etiology

**DOI:** 10.1128/genomeA.00615-17

**Published:** 2017-07-20

**Authors:** Eric Delwart, Beatrix Kapusinszky, Patricia A. Pesavento, Marko Estrada, M. Alexis Seguin, Christian M. Leutenegger

**Affiliations:** a Blood Systems Research Institute, San Francisco, California, USA; b Department of Laboratory Medicine, University of California, San Francisco, California, USA; c Department of Pathology, Microbiology and Immunology, School of Veterinary Medicine, University of California, Davis, California, USA; d IDEXX Laboratories, Inc., West Sacramento, California, USA

## Abstract

We report here the first canine polyomavirus genome, identified by metagenomics in respiratory secretions of two dogs with severe pneumonia, which tested negative for all canine respiratory pathogens except *Mycoplasma cynos*. The isolate, *Canis familiaris* polyomavirus 1 (DogPyV-1), is a beta polyomavirus whose closest known LT antigen relatives are primate polyomaviruses.

## GENOME ANNOUNCEMENT

Three dogs from the same household in the New York City area developed respiratory symptoms in November 2015 following the introduction of a Labrador retriever puppy from a pet store. The puppy fell ill with respiratory signs shortly after joining the household but recovered uneventfully. Two unrelated 10-year-old Dachshunds in the household became sick shortly afterward. One Dachshund died, while the second was diagnosed with pneumonia based on thoracic radiography showing severe diffuse pulmonary infiltrates. Despite intensive antibiotic therapy, this dog was later euthanized due to progressive disease. Lung tissues collected from this dog revealed marked neutrophilic bronchointerstitial pneumonia. Respiratory swab samples from both adult Dachshunds were negative for all respiratory pathogens with the exception of *Mycoplasma cynos* on a real-time PCR panel (Canine Comprehensive Respiratory RealPCRTM Panel, IDEXX, test code 2524; including *Bordetella bronchiseptica*, canine adenovirus type 2, canine distemper virus Quant, canine herpesvirus type 1, canine influenza virus H3N8, H1N1 pandemic influenza virus, H3N2 canine influenza virus, canine parainfluenza virus, canine pneumovirus, canine respiratory coronavirus, *Streptococcus equi* subsp. *zooepidemicus*, and *Mycoplasma cynos*). Additional pathogens tested by PCR included canine circovirus, *Leptospira* spp., *Listeria monocytogenes*, *Pneumocystis carinii*, and *P. murina*. Microbiological cultures on the secretions remained without aerobic growth. 

Enrichment for viral particle-associated nucleic acids in a respiratory swab from the second Dachshund was followed by unbiased nucleic acid amplification using random RT-PCR and use of an Illumina Nextera kit and sequenced using the MiSeq PE300 platform. Similarity sequence searches against all viral protein sequences in GenBank using BLASTx showed the presence of polyomavirus sequences that could be assembled into a typical 5,136-nucleotide-long circular genome for *Canis familiaris* polyomavirus 1 (DogPyV-1) with GenBank accession number KY341899. Retesting of two swabs from the two deceased Dachshunds using a DogPyV-1-specific RT-PCR test confirmed the virus in both respiratory secretions. The presence of DogPyV-1 nucleic acid in lung tissue samples was confirmed using *in situ* hybridization.

In 2016, the taxonomy of the *Polyomaviridae* family was revised to accommodate a rapidly growing number of newly discovered viruses in diverse animal species (1, 2). A new criterion for the creation of novel polyomavirus species is based on >15% difference in sequence identity of the large T antigen (TAg) coding sequence compared to the most closely related species (2). Phylogenetic analysis based on the large TAg indicates that DogPyV-1 is a new species in the genus *Betapolyomavirus* (2). BLASTp analysis of the DogPyV-1 LT antigen showed the most closely related large TAg belonged to different primate polyomaviruses, including *Macaca mulatta* polyomavirus 1 (3), *Cercopithecus erythrotis* polyomavirus 1 (4), Simian virus 12 (5), Vervet monkey polyomavirus 2 (6), human polyomavirus 1 (7), Anubis baboon polyomavius (GenBank accession no. KJ577598.1) and Yellow baboon polyomavirus 2 (6) with identities of 53 to 56% over 89 to 96% of the length of their LT proteins.

The presence of a novel polyomavirus in both tested Dachshunds from the same outbreak raises the possibility that this virus may be a previously unrecognized canine respiratory pathogen. Further studies are under way to determine the virus’s prevalence, cellular tropism, and association with canine respiratory disease.

### Accession number(s).

The full-length viral genomic sequence of DogPyV1 was deposited in GenBank under the accession number KY341899.
